# Effects of different long-term exercise interventions on working memory in children and adolescents: a network meta-analysis

**DOI:** 10.3389/fpsyg.2025.1373824

**Published:** 2025-04-10

**Authors:** Xuejun Guo, Junyu Wang, Jie Liang, Ting Xie, Lin Zhang

**Affiliations:** ^1^Officers College of PAP, Chengdu, China; ^2^School of Exercise and Health, Shanghai University of Sport, Shanghai, China; ^3^Chengdu Xinqiao Primary School, Chengdu, China; ^4^Department of Rehabilitation, West China Hospital Sichuan University Jintang Hospital, Chengdu, China

**Keywords:** children, adolescents, exercise, working memory, network meta-analysis, randomized controlled trials

## Abstract

**Objectives:**

To compare the relative efficacy of different exercise modalities on working memory accuracy and reaction time in healthy children and adolescents.

**Methods:**

A systematic review and network meta-analysis was conducted in accordance with PRISMA guidelines (CRD420251005303). PubMed, Medline, Embase, the Cochrane Central Register of Controlled Trials, and Web of Science were searched from inception to March 1, 2025. Randomized controlled trials examining the impact of any exercise intervention (e.g., aerobic exercise, dance, high-intensity interval training, sports games, mixed exercise) versus control on working memory accuracy and/or reaction time were eligible. Standardized mean differences (SMDs) with 95% confidence intervals (CIs) were calculated, and a random-effects model was applied to account for between-study heterogeneity. The surface under the cumulative ranking curve (SUCRA) was used to determine the relative ranking of each modality.

**Results:**

Thirty-three studies met inclusion criteria for working memory accuracy, and eight studies contributed data on reaction time. Dance demonstrated the highest SUCRA ranking for accuracy (87.8%), and was significantly superior to control (SMD = 0.67, 95% CI 0.13 to 1.21). Aerobic exercise ranked first for reaction time (93.6%), outperforming control (SMD = −0.40, 95% CI −0.69 to −0.11 for accuracy; SMD = −0.82, 95% CI −1.26 to −0.38 for reaction time compared with mixed exercise). Mixed exercise consistently showed lower rankings for both outcomes.

**Conclusion:**

Distinct exercise modalities differentially affect working memory components in young populations. Dance and aerobic exercise appear most beneficial—dance maximizes accuracy, while aerobic exercise optimizes reaction time. Tailoring exercise interventions to specific cognitive targets may enhance working memory development and inform practical, evidence-based strategies in educational and clinical settings.

**Systematic review registration:**

RD420251005303.

## 1 Introduction

Working memory refers to the cognitive system responsible for temporarily storing and manipulating information necessary to perform complex cognitive tasks such as reasoning, decision-making, and problem-solving (Diamond, 2013). It serves as a critical component of executive functions, enabling individuals—particularly children and adolescents—to manage cognitive loads, sustain attention, and adapt flexibly to novel or demanding tasks (Cowan, 2022). During childhood and adolescence, working memory undergoes significant developmental changes, directly influencing academic achievement, language development, and overall cognitive capacity. Insufficient development or deficits in working memory during these formative periods can negatively affect academic performance, leading to difficulties in reading comprehension, mathematics, and information processing, and ultimately impacting long-term educational and occupational outcomes (Hessl et al., 2019; Zhang Y. et al., 2023). Consequently, identifying effective strategies for enhancing working memory in children and adolescents is essential for educators, healthcare professionals, and policymakers concerned with promoting optimal cognitive and developmental trajectories.

A variety of factors contribute to variability in working memory performance among children and adolescents. These factors include genetic predispositions, socioeconomic status, educational quality, nutritional status, and physical activity levels. Among these determinants, physical activity has increasingly been recognized as a modifiable and practical intervention with substantial potential for improving cognitive functioning, including working memory (Xu et al., 2024). Exercise interventions can influence working memory through several biological mechanisms, such as increased neurogenesis, enhanced synaptic plasticity, augmented cerebral blood flow, and improved functional connectivity between brain regions involved in executive functioning (Hatch et al., 2021; Kao et al., 2023; Zhu et al., 2021). Given these neurobiological benefits and ease of implementation, exercise interventions represent a promising, non-pharmacological approach not only for immediate cognitive enhancement but also for fostering lifelong healthy habits in children and adolescents.

Given the accumulating evidence highlighting the cognitive benefits of physical activity, numerous studies have explored the impact of different exercise modalities on working memory in healthy youth. For example, aerobic exercise has consistently demonstrated positive effects on cognitive performance, presumably via enhanced cardiovascular fitness and neurotrophic factor release (VANR et al., 2023; Pickersgill et al., 2022). Similarly, HIIT has been associated with improvements in cognitive processing speed and attentional control, potentially due to the induction of greater neurophysiological adaptations in shorter periods (Yuan et al., 2025). Prior systematic reviews and meta-analyses have generally confirmed the positive effects of exercise on cognitive outcomes; however, these analyses have predominantly focused on comparisons between exercise interventions and non-active control groups (De Greeff et al., 2018; Wang et al., 2023). Thus, there remains a critical knowledge gap regarding the comparative efficacy of various exercise modalities in enhancing working memory specifically. Addressing this gap is essential, as identifying optimal exercise modalities could help maximize cognitive benefits through targeted physical activity interventions.

A network meta-analysis (NMA) offers a robust methodological approach to address this knowledge gap by allowing multiple interventions to be compared in a single, integrated analysis. Unlike traditional pairwise meta-analyses, an NMA enables both direct and indirect comparisons among different exercise modalities and control conditions, yielding a comprehensive ranking of effectiveness (Leucht et al., 2016). Building on this advantage, the present study seeks to synthesize high-quality evidence from randomized controlled trials (RCTs) examining the effects of various exercise interventions on the working memory of healthy children and adolescents. By systematically evaluating these interventions and identifying the most promising exercise types, the findings are expected to inform evidence-based recommendations for exercise programs, guide implementation strategies in educational and community settings, and illuminate avenues for future research.

## 2 Methods

This systematic review and network meta-analysis was conducted in accordance with the 2020 Preferred Reporting Items for Systematic Reviews and Meta-Analyses (PRISMA) and the PRISMA extension statement for network meta-analyses of health care interventions (Hutton et al., 2015; Page et al., 2021). Ethical approval and informed consent were not required, as this study was a meta-analysis of previously published RCTs. The protocol was prospectively registered (CRD420251005303).

### 2.1 Data sources and search

Relevant articles were identified by searching PubMed, Medline, Embase, the Cochrane Central Register of Controlled Trials, and Web of Science from their inception to March 1, 2025. The search strategy combined terms related to “exercise” or “physical activity” (e.g., “aerobic exercise,” “resistance training,” “basketball,” “high-intensity interval training”) and “working memory.” Full details of the database-specific search strategies and term combinations are provided in Supplementary Table 2. In addition to the electronic searches, reference lists of all included studies and bibliographies of systematic reviews published in the past 5 years were screened to identify potentially eligible studies not captured by the initial search. Two independent reviewers (JYW and TX) conducted both title/abstract screening and full-text review in parallel; disagreements were resolved by discussion or by consultation with a third reviewer (XJG).

### 2.2 Eligibility criteria

Studies were included if they met the following criteria: (1) Participants were healthy children (6–12 years) or adolescents (13–18 years); (2) Any type of exercise served as the intervention; (3) Control groups received no intervention, usual care, knowledge-based education, or an alternative exercise type different from that of the experimental group; (4) Intervention duration exceeded four weeks (Kenney et al., 2012); (5) The primary outcome was working memory (i.e., “updating”), measured either by accuracy (higher accuracy indicating better performance) or reaction time (shorter reaction time indicating better performance); (6) The study was a published RCT; and (7) The article was written in English and published in a peer-reviewed journal.

Studies were excluded if they (1) included participants diagnosed with obesity, depression, or attention-deficit/hyperactivity disorder (ADHD), as such conditions might introduce bias; (2) assessed only acute effects of exercise; (3) used multifaceted interventions (e.g., exercise combined with nutritional interventions); (4) did not clearly specify the type of exercise used; or (5) did not report means and standard deviations, and the authors did not respond to data requests within the designated time. Two independent reviewers (JYW and TX) screened potentially relevant articles by assessing the title and abstract, followed by full-text examination, to confirm eligibility. Any disagreement at either stage was resolved through discussion or by consultation with a third reviewer (XJG).

### 2.3 Data extraction

EndNote X9 was used to collate and manage records to avoid duplication. Two reviewers independently extracted information pertaining to publication details (e.g., authors, title, year, journal), participant characteristics (e.g., sample size, age, sex), details of the exercise interventions (duration of a single session, frequency, total intervention duration, exercise modality), and outcome measures (Supplementary Table 3). For effect size calculations, change scores (endpoint minus baseline), standard deviations, and sample sizes were extracted for each group. When change scores or standard deviations were not directly available, they were derived or converted following guidelines in the Cochrane Handbook (Higgins and Green, 2008). If relevant data were incomplete, at least four attempts over 6 weeks were made to contact the corresponding authors.

### 2.4 Data coding

For the purpose of making structured comparisons of different exercise modalities in the network meta-analysis, exercise interventions were coded into six categories: “aerobic exercise (AE),” “dance (DC),” “mixed exercise (ME),” “high-intensity interval training (HIIT),” “sports games (SG),” and “CON” for controls. Specific definitions and examples of each exercise modality are detailed in Supplementary Table 3.

### 2.5 Risk of Bias assessment

Two reviewers independently evaluated the risk of bias at the study level using the revised Cochrane risk of bias tool (RoB 2) (Sterne et al., 2019). This tool assesses risk of bias across the following domains: (1) randomization process, (2) deviations from intended interventions, (3) missing outcome data, (4) measurement of the outcome, and (5) selection of the reported results. Any disagreements in judgments were resolved through consultation with a third reviewer.

### 2.6 Statistical analysis

Statistical analyses were performed using Stata software (version 17.0, StataCorp LLC, College Station, TX, United States). A network meta-analysis approach was used to compare the effects of different exercise interventions on working memory outcomes, assuming all included interventions could influence the results. A network plot was generated to illustrate the connections among treatment comparisons, ensuring feasibility of the network structure. Given the anticipated clinical heterogeneity, a random-effects model was applied to account for both within- and between-study variability.

Because the measures of updating varied across studies in terms of scales or units, standardized mean differences (SMD) with 95% confidence intervals (CIs) were calculated. Heterogeneity was evaluated using the *I*^2^ statistic, where thresholds of 25, 50, and 75% represent low, moderate, and high heterogeneity, respectively. The “network” and “mvmeta” packages in Stata’s Bayesian framework were applied to facilitate the network meta-analysis. The relative efficacy of each intervention was ranked according to the surface under the cumulative ranking (SUCRA) curves; a higher SUCRA value indicates a more favorable treatment effect.

To assess publication bias, adjusted funnel plots were generated, and Egger’s test was performed; a *p*-value <0.05 indicated potential publication bias (Chaimani et al., 2013). Prediction interval plots were also used to explore heterogeneity and interpret variability in effect sizes. All tests were two-sided, and *p*-values <0.05 were considered statistically significant.

## 3 Results

### 3.1 Characteristics of the included studies

**Figure 1:**
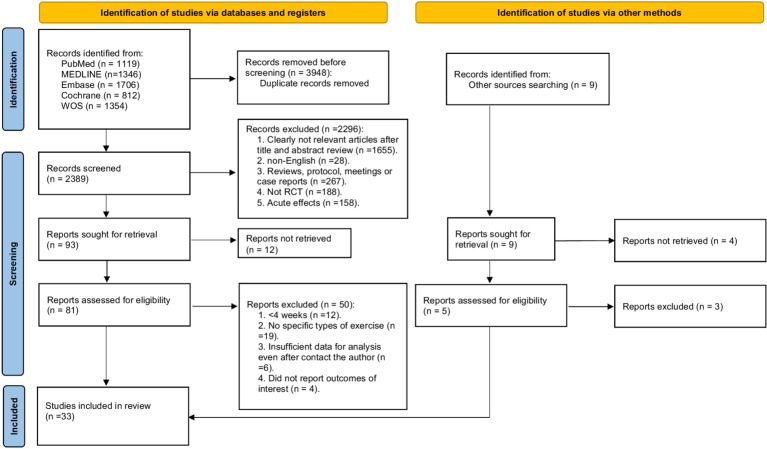
PRISMA Flow diagram of the search process for studies.

A total of 6,337 records were initially identified through electronic database searches. After removing 3,948 duplicates, 2,389 articles were screened by title and abstract, and 2,296 were excluded based on relevance. Ultimately, 81 full-text articles were assessed for eligibility, leading to the inclusion of 33 RCTs comprising 20,697 healthy children and adolescents for the systematic review and network meta-analysis (Figure 1; Alesi et al., 2016; Beck et al., 2016; Contreras-Osorio et al., 2022; De Bruijn et al., 2021; Egger et al., 2019; Gentile et al., 2020; Jeon and Ha, 2017; Kamijo et al., 2011; Knatauskaitė et al., 2021; Koutsandréou et al., 2016; Kvalø et al., 2017; Latino et al., 2021; Leahy et al., 2020; Leong et al., 2015; Lind et al., 2018; Lubans et al., 2020; Ludyga et al., 2018; Mavilidi et al., 2020; Meijer et al., 2022; Meijer et al., 2020; Mora-Gonzalez et al., 2024; Park and Jee, 2022; Robinson et al., 2022; Schmidt et al., 2015; St Laurent et al., 2019; Tocci et al., 2022; Torbeyns et al., 2017; Tottori et al., 2019; Van den Berg et al., 2019; Veldman et al., 2020; Wassenaar et al., 2021; Zhang M. et al., 2023; Zinelabidine et al., 2022). Detailed characteristics of the included studies are presented in Supplementary Table 4.

All included RCTs were published between 2011 and 2023, with a median publication year of 2020. Sample sizes ranged from 33 to 16,017 participants (median 73). The mean age of participants varied between 6.2 and 16.2 years, with a median of 10.0 years. Regarding the exercise interventions, 13 studies employed AE, 4 implemented DC, 6 utilized HIIT, 10 incorporated ME, 10 involved SG, and 32 studies used regular physical education or similar activities as the CON. Single-session durations ranged from 4 to 80 min (median 30 min), the frequency of exercise varied from once weekly to five times per week (median three times weekly), and the total intervention duration ranged from 4 to 40 weeks (median 12 weeks).

### 3.2 The results of network meta-analysis

#### 3.2.1 Working memory accuracy

**Figure 2:**
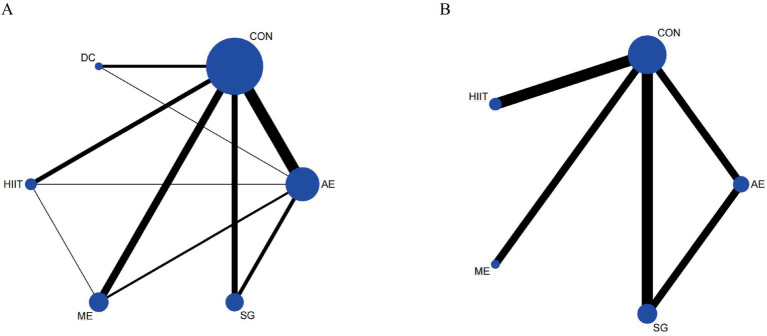
Network of eligible treatment comparisons for outcomes. **(A)** Working memory accuracy, **(B)** Working memory reaction time.

**Figure 3:**
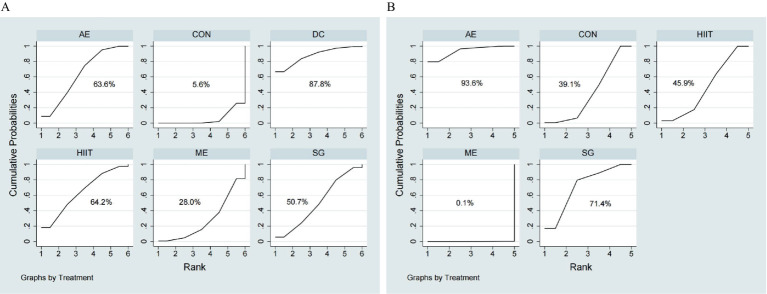
Ranking of exercise strategies based on probability of their effects for outcomes. **(A)** Working memory accuracy, **(B)** Working memory reaction time.

A network meta-analysis of 33 RCTs (20,697 children and adolescents) evaluated the impact of various exercise modalities on working memory accuracy. Figure 2A illustrates the direct comparisons and sample size distributions among interventions. According to the surface under the cumulative ranking (SUCRA) probabilities (Figure 3A), the top three interventions for improving working memory accuracy were dance (DC, 87.8%), high-intensity interval training (HIIT, 64.2%), and aerobic exercise (AE, 63.6%), whereas the lowest-ranked modality was the control condition (CON).

As shown in Table 1, DC (SMD = 0.67, 95% CI 0.13 to 1.21) and AE (SMD = 0.40, 95% CI 0.11 to 0.69) significantly enhanced working memory accuracy compared with CON.

**Table 1:** League table of outcomes.

Working memory accuracy					
DC					
0.25 (-0.44,0.94)	HIIT				
0.27 (-0.33,0.86)	0.02 (-0.49,0.52)	AE			
0.35 (-0.30,1.00)	0.10 (-0.46,0.67)	0.08 (-0.31,0.48)	SG		
0.52 (-0.12,1.15)	0.27 (-0.26,0.80)	0.25 (-0.16,0.66)	0.17 (-0.32,0.65)	ME	
**0.67 (0.13,1.21)**	0.42 (-0.01,0.85)	**0.40 (0.11,0.69)**	0.32 (-0.05,0.69)	0.15 (-0.19,0.49)	CON

Working memory reaction time				
AE				
-0.17 (-0.53,0.19)	SG			
-0.34 (-0.72,0.04)	-0.17 (-0.55,0.21)	HIIT		
-0.36 (-0.71,-0.00)	-0.19 (-0.52,0.14)	-0.02 (-0.17,0.13)	CON	
**-0.82 (-1.26,-0.38)**	**-0.66 (-1.06,-0.25)**	**-0.49 (-0.80,-0.17)**	**-0.46 (-0.72,-0.21)**	ME

Bold values indicate statistically significant differences (*p* < 0.05).

#### 3.2.2 Working memory reaction time

A network meta-analysis of 8 RCTs (17,940 children and adolescents) evaluated the effects of different exercise modalities on working memory reaction time. Figure 2B depicts the direct comparisons and sample size distributions among interventions. Based on SUCRA (Figure 3B), the top three interventions for reducing working memory reaction time were AE (93.6%), SG (71.4%), and HIIT (45.9%), while mixed exercise (ME) demonstrated the lowest ranking (5.6%).

Table 1 shows that, compared with ME, all other modalities produced a significantly greater reduction in working memory reaction time. Specifically, relative to ME, AE (SMD = −0.82, 95% CI −1.26 to −0.38), SG (SMD = −0.66, 95% CI −1.06 to −0.25), HIIT (SMD = −0.49, 95% CI −0.80 to −0.17), and CON (SMD = −0.46, 95% CI −0.72 to −0.21) were all associated with significantly improved (i.e., shortened) reaction times.

### 3.3 Risk of Bias and publication Bias

Among the 33 RCTs, 17 were judged as low overall risk of bias, 13 showed some concerns, and 3 were rated as high risk. Regarding the randomization process, 27 trials exhibited low risk, and 6 had some concerns. For deviations from intended interventions, 29 trials had low risk, and 4 had some concerns. In terms of missing outcome data, 24 trials exhibited low risk, 7 had some concerns, and 2 were considered high risk. With respect to outcome measurement, 32 were rated as low risk, and 1 presented some concerns. Finally, for selective reporting, 31 were rated as low risk, 1 showed some concerns, and 1 was deemed high risk (Supplementary Table 5).

Funnel plots were used to examine potential publication bias (Supplementary Table 6). The scatter distribution around the vertical axis showed varying degrees of asymmetry, indicating the possibility of publication bias. In particular, Supplementary Figures 1A,B demonstrated notable asymmetry. However, Egger’s tests for both working memory accuracy and working memory reaction time produced *p*-values greater than 0.05, suggesting no statistically significant evidence of publication bias in the overall analysis.

## 4 Discussion

This network meta-analysis integrated data from 33 RCTs involving 20,697 healthy children and adolescents to compare the effects of various exercise interventions on working memory. Several noteworthy findings emerged. First, DC was identified as the most effective intervention for improving working memory accuracy, outperforming other exercise modalities in this domain. Second, AE demonstrated the greatest efficacy in enhancing working memory reaction time and also showed a significant benefit in accuracy compared with the control condition. Third, ME ranked lower for both working memory accuracy and reaction time. Taken together, these observations underscore the nuanced effects of different exercise modalities on distinct facets of working memory and highlight the potential value of carefully selecting specific types of exercise interventions to optimize cognitive outcomes among healthy children and adolescents.

Working memory accuracy is crucial for children and adolescents because it enables them to efficiently process, store, and retrieve information in real time, facilitating a wide range of academic and everyday tasks (Diamond, 2013). Although working memory continues to develop throughout adolescence, many young individuals exhibit variability in their capacity to handle cognitively demanding activities, underscoring the importance of interventions that can strengthen these foundational skills (Xue et al., 2019). In the present study, dance emerged as the top-ranked modality for enhancing working memory accuracy among all examined interventions, reinforcing the value of structured, skill-based physical activities in supporting core cognitive functions. This finding largely aligns with earlier studies that reported moderate-to-strong cognitive gains from dance-related programs but departs from research that highlighted aerobic training as the most potent approach for improving executive functions (Burzynska et al., 2017a; May et al., 2021). The distinctive value of dance for children and adolescents may stem from its unique combination of rhythmic movement, coordination of motor skills, and need for sustained attention, all of which can engage sensory, motor, and cognitive systems simultaneously (Mansfield et al., 2018). From a mechanistic perspective, dance may optimize neural circuitry underlying executive functions by promoting neuroplastic changes through complex motor sequencing, fostering social and emotional engagement, and requiring continuous cognitive monitoring of posture, balance, and spatial awareness (Burzynska et al., 2017b). Consequently, this multifaceted stimulus could lead to more pronounced improvements in working memory accuracy than activities focusing predominantly on endurance, strength, or simpler motor tasks.

In addition to accuracy, working memory reaction time provides critical insight into how swiftly children and adolescents can process and respond to cognitive demands. The present study identified aerobic exercise as the top-ranked modality for improving reaction time, and it also yielded notable benefits for working memory accuracy. This aligns with several previous investigations that have highlighted the cognitive advantages of endurance-based activities, although some meta-analyses have reported mixed findings depending on factors such as intervention duration and participant age (Liang et al., 2021). Aerobic exercise, encompassing activities like running, cycling, and swimming, is often more accessible and feasible for children and adolescents in both formal (e.g., school-based programs) and informal (e.g., community or home-based) settings, thereby enhancing its practical appeal. From a mechanistic perspective, aerobic exercise may facilitate improvements in working memory through augmented cerebral blood flow, release of neurotrophic factors (e.g., brain-derived neurotrophic factor), and enhanced neuroplasticity, all of which contribute to more efficient neural processing (Walsh et al., 2020). The repetitive and rhythmic nature of aerobic activities can also support attentional control and self-regulation, enabling individuals to sustain focus over extended periods. Consequently, by simultaneously promoting neural health and fostering key cognitive processes, aerobic exercise is uniquely positioned to exert robust improvements on both the speed and accuracy dimensions of working memory in children and adolescents.

Interestingly, this study revealed that ME ranked relatively lower for both working memory accuracy and reaction time. Among children and adolescents, ME typically involves combining multiple exercise modalities into a single session, such as initiating with aerobic warm-up, transitioning into resistance exercises like squats and lunges, and culminating in sports games focused on skill development and teamwork. While in theory, such multi-component sessions could offer diverse physiological and cognitive stimuli, the present results suggest that this versatility may inadvertently dilute the specific training effects necessary for enhancing core cognitive processes. One possible explanation is that each component of a mixed session, being relatively short, might not reach the threshold intensity or consistency needed to elicit robust neurocognitive adaptations (Costigan et al., 2016). In addition, rapid transitions between disparate activities could impose competing demands on attentional resources, reducing the sustained engagement crucial for working memory improvement. Moreover, heterogeneous protocols classified under the ME label in existing studies may further contribute to inconsistent outcomes, as the intensity, duration, and sequencing of different activities can vary considerably (Mezcua-Hidalgo et al., 2019). Collectively, these factors may lessen the impact of mixed exercise interventions on working memory in comparison to more targeted programs such as dance or aerobic exercise, emphasizing the importance of structured and purposefully designed physical activity regimens for promoting optimal cognitive benefits in young populations.

These findings bear considerable practical significance for educational programs, public health strategies, and clinical interventions aimed at enhancing cognitive development in children and adolescents. By highlighting the relative effectiveness of distinct exercise modalities—particularly dance and aerobic exercise—this study provides an evidence-based framework to inform physical education curricula, after-school activity programs, and structured exercise interventions. Encouraging youth to engage in targeted exercise routines aligned with these proven modalities may not only enhance working memory functions but also cultivate enduring physical activity habits, thereby fostering both cognitive and physical health benefits over the long term (Wang et al., 2025). Moreover, these insights can help policymakers and practitioners allocate resources efficiently, designing accessible and scalable exercise interventions that align with the developmental needs of diverse populations. Through careful integration of scientifically grounded exercise programs into educational and community settings, stakeholders can bolster working memory development and ultimately support the broader academic and psychosocial well-being of children and adolescents.

This study presents several noteworthy strengths and limitations. First, one of its key strengths lies in the application of a network meta-analysis framework, which allowed for simultaneous comparisons among multiple exercise modalities using both direct and indirect evidence. This comprehensive approach enhances the robustness of the findings by integrating data across a diverse range of randomized controlled trials. Second, the large aggregated sample size of the included studies, encompassing varied geographic locations and participant characteristics, helps bolster the external validity and generalizability of the results. Nevertheless, a few limitations warrant consideration. First, heterogeneity in intervention protocols—for instance, disparities in session duration, frequency, and intensity within the same exercise category—may have influenced effect estimates and constrained the capacity to draw definitive conclusions regarding optimal parameters. Second, variations in outcome measures and assessments of working memory accuracy and reaction time across different trials may introduce measurement bias and limit the direct comparability of results. Finally, although the risk of bias was carefully appraised, potential biases within the primary studies, such as incomplete blinding or selective reporting, cannot be entirely ruled out. These factors should be taken into account when interpreting the findings and underscore the need for more standardized and methodologically rigorous research to elucidate the full impact of distinct exercise interventions on working memory in children and adolescents.

## 5 Conclusion

This network meta-analysis of 33 RCTs with 20,697 healthy children and adolescents reveals that distinct exercise modalities yield different cognitive benefits. Dance is most effective for enhancing working memory accuracy, whereas aerobic exercise confers the greatest improvement in reaction time. Other interventions, including high-intensity interval training and sports games, demonstrate intermediate benefits, whereas mixed exercise shows comparatively limited effects. These findings underscore the importance of aligning exercise modalities with specific cognitive targets when developing educational and clinical interventions. Tailored exercise programs that match the modality to a particular working memory outcome may optimize cognitive gains and foster long-term healthy habits. Future research should refine intervention protocols, investigate underlying mechanisms, and explore applicability across diverse pediatric populations to promote evidence-based strategies for enhancing working memory and broader cognitive development.

## Data Availability

The original contributions presented in the study are included in the article/Supplementary material, further inquiries can be directed to the corresponding author/s.
