# Supplementary material for: Effects of different long-term exercise interventions on working memory in children and adolescents: a network meta-analysis
Source: Front Psychol. 2025 Apr 10;16:1373824. doi: 10.3389/fpsyg.2025.1373824 (PMC12019642; doi:10.3389/fpsyg.2025.1373824)

3.1 Working Memory Accuracy

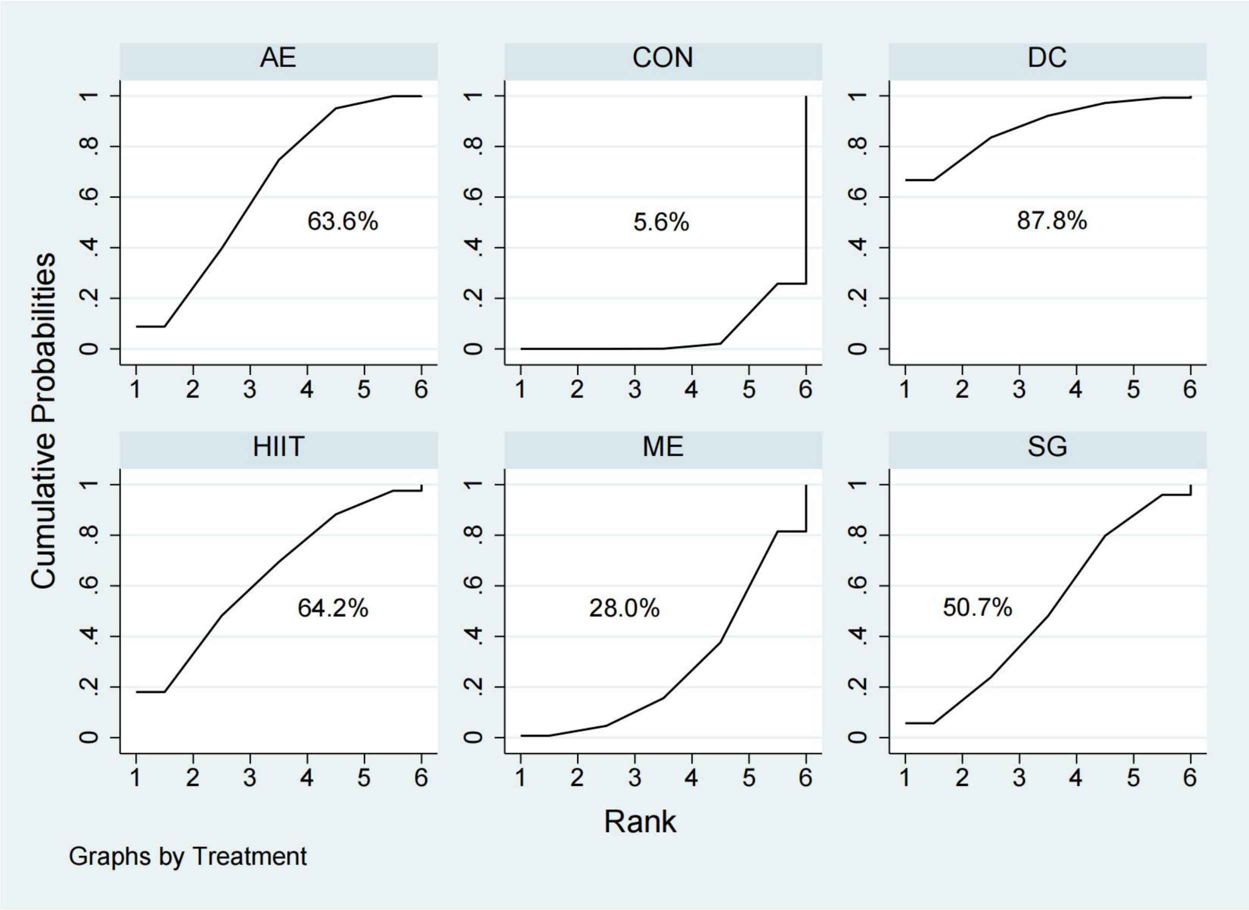

3.2 Working Memory Reaction Time

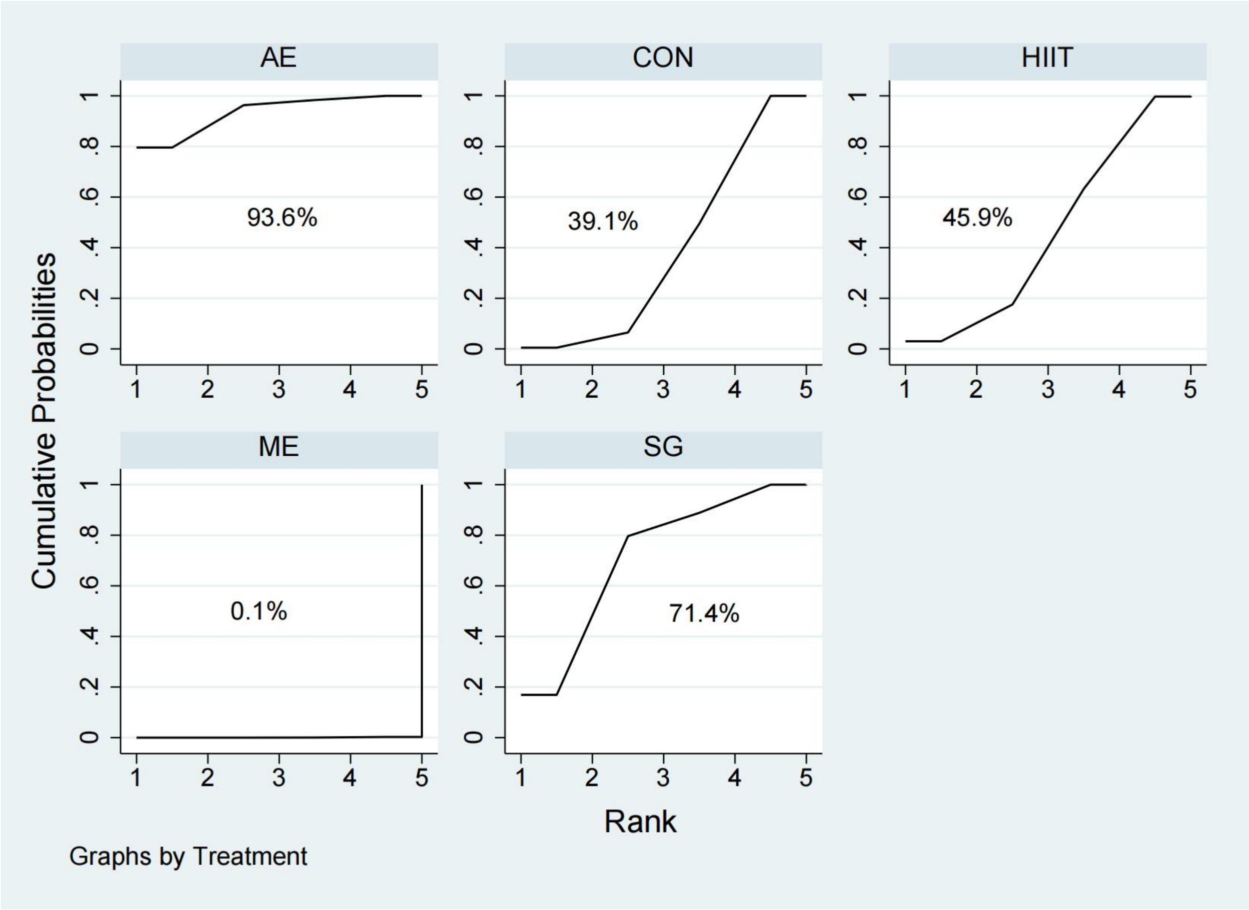

Supplement: Supplementary file 1 [file Image_1.pdf]
