# Supplementary material for: Effects of different long-term exercise interventions on working memory in children and adolescents: a network meta-analysis
Source: Front Psychol. 2025 Apr 10;16:1373824. doi: 10.3389/fpsyg.2025.1373824 (PMC12019642; doi:10.3389/fpsyg.2025.1373824)
Supplement: Supplementary file 2 [file Data_Sheet_1.pdf]

Supplementary

Table of Contents:

Supplementary 1: PRISMA Checklist **1**

Supplementary 2: Search Strategy **5**

Supplementary 3: Definitions of exercise types and non-exercise training control **7**

Supplementary 4: Characteristics of studies and subjects included in the review **22**

Supplementary 5: Risk of Bias **25**

Supplementary 6: Publication bias **22**

Supplementary 1: PRISMA Checklist

Item #	Checklist Item	Reported on Page #
		1
1	Identify the report as a systematic review incorporating a network meta-analysis (or related form of meta-analysis).	1
2	Provide a structured summary including, as applicable:<br>**Background:** main objectives<br>**Methods:** data sources; study eligibility criteria, participants, and interventions; study appraisal; and *synthesis methods, such as network meta-analysis.*<br>**Results:** number of studies and participants identified; summary estimates with corresponding confidence/credible intervals; treatment rankings may also be discussed. Authors may choose to summarize pairwise comparisons against a chosen treatment included in their analyses for brevity.<br>**Discussion/Conclusions:** limitations; conclusions and implications of findings.<br>**Other:** primary source of funding; systematic review registration number with registry name.	1
3	Describe the rationale for the review in the context of what is already known*, including mention of why a network meta-analysis has been conducted.*_	2-3
4	Provide an explicit statement of questions being addressed, with reference to participants, interventions, comparisons, outcomes, and study design (PICOS).	3-4
5	Indicate whether a review protocol exists and if and where it can be accessed (e.g., Web address); and, if available, provide registration information, including registration number.	1
6	Specify study characteristics (e.g., PICOS, length of follow-up) and report characteristics (e.g., years considered, language, publication status) used as criteria for eligibility, giving rationale. *Clearly describe eligible treatments included in the treatment network, and note whether any have been clustered or merged into the same node (with justification).*_	3-4
7	Describe all information sources (e.g., databases with dates of coverage, contact with study authors to identify additional studies) in the search and date last searched.	3-4
8	Present full electronic search strategy for at least one database, including any limits used, such that it could be repeated.	Supplementary 2
9	State the process for selecting studies (i.e., screening, eligibility, included in systematic review, and, if applicable, included in the meta-analysis).	4, Figure 3
10	Describe method of data extraction from reports (e.g., piloted forms, independently, in duplicate) and any processes for obtaining and confirming data from investigators.	3-4

11	List and define all variables for which data were sought (e.g., PICOS, funding sources) and any assumptions and simplifications made.	3-4
S1	Describe methods used to explore the geometry of the treatment network under study and potential biases related to it. This should include how the evidence base has been graphically summarized for presentation, and what characteristics were compiled and used to describe the evidence base to readers.	4, Supplementary 3
12	Describe methods used for assessing risk of bias of individual studies (including specification of whether this was done at the study or outcome level), and how this information is to be used in any data synthesis.	4, Supplementary 5
13	State the principal summary measures (e.g., risk ratio, difference in means). Also describe the use of additional summary measures assessed, such as treatment rankings and surface under the cumulative ranking curve (SUCRA) values, as well as modified approaches used to present summary findings from meta-analyses.	4
14	Describe the methods of handling data and combining results of studies for each network meta-analysis. This should include, but not be limited to:<br>• Handling of multi-arm trials;<br>• Selection of variance structure;<br>• Selection of prior distributions in Bayesian analyses; and<br>• Assessment of model fit.	4
S2	Describe the statistical methods used to evaluate the agreement of direct and indirect evidence in the treatment network(s) studied. Describe efforts taken to address its presence when found.	4
15	Specify any assessment of risk of bias that may affect the cumulative evidence (e.g., publication bias, selective reporting within studies).	4
16	Describe methods of additional analyses if done, indicating which were pre-specified. This may include, but not be limited to, the following:<br>• Sensitivity or subgroup analyses;<br>• Meta-regression analyses;<br>• Alternative formulations of the treatment network; and<br>• Use of alternative prior distributions for Bayesian analyses (if applicable)._	NA
17	Give numbers of studies screened, assessed for eligibility, and included in the review, with reasons for exclusions at each stage, ideally with a flow diagram.	Figure 1
S3	Provide a network graph of the included studies to enable visualization of the geometry of the treatment network.	Figure 2
S4	Provide a brief overview of characteristics of the treatment	Supplementary

	network. This may include commentary on the abundance of trials and randomized patients for the different interventions and pairwise comparisons in the network, gaps of evidence in the treatment network, and potential biases reflected by the network structure.	3
18	For each study, present characteristics for which data were extracted (e.g., study size, PICOS, follow-up period) and provide the citations.	4-5, Supplementary 4
19	Present data on risk of bias of each study and, if available, any outcome level assessment.	Figure 2, and Supplementary 5
20	For all outcomes considered (benefits or harms), present, for each study: 1) simple summary data for each intervention group, and 2) effect estimates and confidence intervals. *Modified approaches may be needed to deal with information from larger networks.*	5, Table 1
21	Present results of each meta-analysis done, including confidence/credible intervals. In larger networks, authors may focus on comparisons versus a particular comparator (e.g. placebo or standard care), with full findings presented in an appendix. League tables and forest plots may be considered to summarize pairwise comparisons. If additional summary measures were explored (such as treatment rankings), these should also be presented.	4-5, table 1
S5	Describe results from investigations of inconsistency. This may include such information as measures of model fit to compare consistency and inconsistency models, *P* values from statistical tests, or summary of inconsistency estimates from different parts of the treatment network.	5-6
22	Present results of any assessment of risk of bias across studies for the evidence base being studied.	5-6, Supplementary 5
23	Give results of additional analyses, if done (e.g., sensitivity or subgroup analyses, meta-regression analyses*, alternative network geometries studied, alternative choice of prior distributions for Bayesian analyses,* and so forth).	NA
24	Summarize the main findings, including the strength of evidence for each main outcome; consider their relevance to key groups (e.g., healthcare providers, users, and policy-makers).	6-8
25	Discuss limitations at study and outcome level (e.g., risk of bias), and at review level (e.g., incomplete retrieval of identified research, reporting bias). *Comment on the validity of the assumptions, such as transitivity and consistency. Comment on any concerns regarding network geometry (e.g., avoidance of certain comparisons).*	6-8
26	Provide a general interpretation of the results in the context of	7-8

	other evidence, and implications for future research.	
27	Describe sources of funding for the systematic review and other support (e.g., supply of data); role of funders for the systematic review. This should also include information regarding whether funding has been received from manufacturers of treatments in the network and/or whether some of the authors are content experts with professional conflicts of interest that could affect use of treatments in the network.	8-9

PICOS = population, intervention, comparators, outcomes, study design.

Supplementary 2: Search Strategy

Search Strategy:

Search number	Query
17	((((((((((child[MeSH Terms]) OR (adolescent[MeSH Terms])) OR (children[Title/Abstract])) OR (adolescen*[Title/Abstract])) OR (pupils[Title/Abstract])) OR (preadolescent[Title/Abstract])) OR (youth[Title/Abstract])) OR (juvenile[Title/Abstract])) OR (teenager*[Title/Abstract])) AND ((((((((exercise[MeSH Terms]) OR ("physical activity"[Title/Abstract])) OR (training[Title/Abstract])) OR (workout[Title/Abstract])) OR (sport*[Title/Abstract])) OR (jogging[Title/Abstract])) OR ("resistance training"[Title/Abstract])) OR ("aerobic exercise"[Title/Abstract]))) AND ((((executive function[MeSH Terms]) OR ("executive function*"[Title/Abstract])) OR ("working memory"[Title/Abstract])) OR (updating[Title/Abstract])) AND ((randomized controlled trial[pt] OR controlled clinical trial[pt] OR randomized[tiab] OR placebo[tiab] OR drug therapy[sh] OR randomly[tiab] OR trial[tiab] OR groups[tiab]) NOT (animals[mh] NOT humans[mh]))
16	(randomized controlled trial[pt] OR controlled clinical trial[pt] OR randomized[tiab] OR placebo[tiab] OR drug therapy[sh] OR randomly[tiab] OR trial[tiab] OR groups[tiab]) NOT (animals[mh] NOT humans[mh])
15	humans[mh]
14	animals[mh]
13	randomized controlled trial[pt] OR controlled clinical trial[pt] OR randomized[tiab] OR placebo[tiab] OR drug therapy[sh] OR randomly[tiab] OR trial[tiab] OR groups[tiab]
12	(executive function[MeSH Terms]) OR ("executive function*"[Title/Abstract])) OR ("working memory"[Title/Abstract])) OR (updating[Title/Abstract])
11	("ex("working memory"[Title/Abstract]) OR (updating[Title/Abstract])
10	executive function[MeSH Terms]
9	(((((((exercise[MeSH Terms]) OR (resistance training[MeSH Terms]) OR (Yoga[MeSH Terms]) OR ("physical activity"[Title/Abstract])) OR (training[Title/Abstract])) OR (workout[Title/Abstract])) OR (sport*[Title/Abstract])) OR (jogging[Title/Abstract])) OR ("resistance training"[Title/Abstract])) OR ("aerobic exercise"[Title/Abstract])
8	("physical activity"[Title/Abstract]) OR (training[Title/Abstract]) OR (workout[Title/Abstract]) OR (sport*[Title/Abstract]) OR (jogging[Title/Abstract]) OR ("resistance training"[Title/Abstract]) OR ("aerobic exercise"[Title/Abstract])
7	Yoga[MeSH Terms]
6	Resistance training[MeSH Terms]
5	(exercise[MeSH Terms]
4	((((((((child[MeSH Terms]) OR (adolescent[MeSH Terms])) OR (children[Title/Abstract])) OR (adolescen*[Title/Abstract])) OR (pupils[Title/Abstract])) OR (preadolescent[Title/Abstract])) OR (youth[Title/Abstract])) OR (juvenile[Title/Abstract])) OR (teenager*[Title/Abstract])

3	(children[Title/Abstract]) OR (adolescen*[Title/Abstract]) OR (pupils[Title/Abstract]) OR (preadolescent[Title/Abstract]) OR (youth[Title/Abstract]) OR (juvenile[Title/Abstract]) OR (teenager*[Title/Abstract])
2	Adolescent[MeSH Terms]
1	Child[MeSH Terms]

Supplementary file 3: Definitions of exercise types and non-exercise training control

Abbreviation	Full name	Definitions	Examples
AE	Aerobic Exercise	Aerobic exercise aims to enhance cardiorespiratory function through sustained, rhythmic, and moderate-intensity physical activities. Predominantly reliant on oxygen for energy production, these exercises typically have an extended duration[1].	Jogging, jumping rope, relay races, treadmill training and circuit training, etc.
CON	Control Group	Regular physical activity or cognitive education with no additional physical activity interventions.	Regular teaching programs on physical activity, or problem-solving exercises, memory enhancement training programs and interactive learning modules to enhance cognitive functioning and promote peer interaction.
DC	Dance	Dance constitutes a choreographed form of physical activity, synchronized to musical rhythms and compositions.	Aerobic dancing to specific rhythmic patterns.
HIIT	High-Intensity Interval Training	HIIT involves either (a) short or long intervals of high intensity exercise interspersed by short rest periods, or (b) reoccurring short or long bouts of maximal sprints, interspersed by a prolonged rest period between exercises[3].	Sprinting, where you run at maximum speed for 30 seconds and then rest for 30 seconds.
ME	Mixed Exercise	Mixed exercise integrates diverse modalities of physical activity, encompassing cardiovascular training, resistance exercises, and various game-based activities like ball games, to offer a well-rounded workout regimen. This multifaceted approach is frequently employed in cross-training programs to optimize fitness outcomes.	Starting with a cardio warm-up, followed by a series of resistance exercises such as squats and lunges, and concluding with sports games to develop skills and team coordination.

Abbreviation	Full name	Definitions	Examples
SG	Sports Games	Sports games characterized by specific rules, objectives and skills required. These activities typically include skill instruction, practice sessions, organized sports games and small, non-competitive matches.	Football-based strategic maneuvers and basketball-centric passing exercises, etc.

Reference

1. Thompson PD, Arena R, Riebe D, Pescatello LS. ACSM's new preparticipation health screening recommendations from ACSM's guidelines for exercise testing and prescription, ninth edition. Current sports medicine reports. 2013;12(4):215-217.
2. Erickson KI, Hillman C, Stillman CM, et al. Physical Activity, Cognition, and Brain Outcomes: A Review of the 2018 Physical Activity Guidelines. Medicine and science in sports and exercise. 2019;51(6):1242-1251.
3. Buchheit M, Laursen PB. High-intensity interval training, solutions to the programming puzzle: Part I: cardiopulmonary emphasis. Sports medicine (Auckland, NZ). 2013;43(5):313-338.

Supplementary 4: Characteristics of studies and subjects included in the review

Study	Subjects (intervention/ control)	Sex (boy/girl) (intervention/ control)	Mean age (intervention/ control)	BMI (intervention / control)	Intervention detail		Intervention site	Intervention/control of intensity	Session duration	Training frequency	Duration	Outcomes (accuracy)	Outcomes (reaction time)
					Intervention group	Control group							
Alesi et al. (2016)	44 (24/20)	24/0 vs. 20/0	8.8±1.1 vs. 9.3±0.9	19.2±4.5 vs. 21.5±4.7	ME: Soccer training, including individual skills, technique or/and one-on-one situations, opposed games involving three-on-three and five-on-five	Usually activities	Outdoor Playground	N/A	60 min	2 × per week	6 months	Postintervention, Digit Span Test	
Beck et al. (2016)	102 (55/57)	30/25 vs. 28/29	7.5±0.02 vs. 7.5±0.02	16.5±0.3 vs. 16.5±0.3	ME: Includes coordination, aerobic and static movements for jumping, climbing, hopping, throwing, and balancing on one foot.	Usually activities	Indoor Classroom	60-85% HRmax	60 min	5 × per week	6 weeks	Postintervention, Spatial Span	
Contreras-Osorio et al. (2022)	90 (30/30/30)	16/14 vs. 16/14 vs. 15/15	11.4±0.6 vs. 11.4±0.9 vs.11.5±0.6	19.3±2.4 vs. 19.2±3.1 vs. 19.3 ± 3.7	(a) ME: Individual improvement tasks, one-phase improvement tasks, combination tasks, and real play. (b) AE: Athletic abilities with a technical component,	Usually activities	Outdoor Playground	Borg scale (CR-10) (5-7)	40 min	2 × per week	12 weeks	Postintervention, Verbal fluency	

					lifts and repetitions through the set, introduction to fartlek, and continuous running.								
de Bruijn et al. (2021)	62 (22/23/17)	11/11 vs. 12/11 vs. 5/12	9.2±0.7 vs. 9.0±0.6 vs. 9.4±0.6	N/A	(a) AE: Repetitive and automated skills, for example running, relays, or individualized exercises such as jumping jacks, planks, or squats.<br>(b) SG: Throwing, catching, balancing, and dodging balls	Regular program	N/A	N/A	30 min	4 × per week	14 weeks		Postintervention, Digit Span Test
Egger et al. (2019)	142 (49/47/46)	21/28 vs. 21/26 vs. 22/24	7.9±0.4 vs. 7.9±0.4 vs. 7.8±0.4	16.51±2.91 vs. 16.21±2.22 vs. 16.21±2.36	(a) AE: Mimic the teacher in aerobic movements such as horse racing and jumping.<br>(b) SG: Standing in a circle and playing the game “Horserace”.	Cognitive activities without PA	Outdoor Playground	N/A	10 min	2 × per day, 5 × per week	20 weeks	Postintervention, Backwards Colour Recall task	
Gentile et al. (2020)	411 (211/200)	197 vs. 214	9.6±1.8 vs. 9.6±1.8	N/A	ME: Movements are completed through instructions and include a variety of elements such as coordination, aerobic and physical play.	Standard PE lessons	Indoor Sports Center	N/A	25 min	5 × per week	14 weeks	Postintervention, Digit Span Test	
Jeon et al.	40	10/0 vs. 10/0 vs.	15.06±0.73	N/A	(a) AE: 40% VO2Max	Stretchin	N/A	Average	(a)	4 × per	12 weeks	Postintervent	

(2017)	(10/10/10/10)	10/0 vs. 10/0	vs. 15.47 ± 0.78 vs. 15.15 ± 0.33 vs. 15.05 ± 0.41		aerobic exercises (b) AE: 55% VO2Max aerobic exercises (c) AE: 70% VO2Max aerobic exercises	g		heart rate (AE1:40%VO2max, AE2: 55%VO2max, AE3: 70%VO2max)	AE:43.34 min (b) AE: 33.33 min (c) AE: 25.76 min	week		ion, Wechsler intelligence scale	
Kamijo et al. (2011)	36 (20/16)	9/11 vs. 8/8	8.9±0.5 vs. 9.1±0.6	20.5±3.8 vs. 20.7±5.2	ME: Aerobic exercise, resistance training, motor skill acquisition.	Usually activities	Outdoor Playground	Moderate-to-vigorous intensity	70 min	5 × per week	9 months	Postintervention, Sternberg task	
Knatauskaitė et al. (2021)	135 (48/41/46)	67 vs. 68	13.0±0.7 vs. 13.0±1.5 vs. 12.0±0.7	N/A	(a) AE: Repetitive exercises or games that focus on aerobic endurance, such as running or relay running. (b) SG: Playful exercise games in coordination such as balance, bilateral coordination, and spatial orientation.	Assisted homework sessions	Outdoor Playground	64-76% of maximum heart rate	45 min	3 × per week	10 weeks	Postintervention, Matching grids task	Postintervention, Matching grids task
Koutsandréou et al.	71 (27/23/21)	32 vs. 39	9.4±0.6	N/A	(a) AE: Running-based games at	Assisted homewor	Outdoor Playgrou	Average heart	45 min	3 × per week	10 weeks	Postintervention, Letter	

(2016)					moderate-to-vigorous intensity;<br>(b) ME: Playful exercises in coordination such as balance, bilateral coordination, and spatial orientation.	k sessions	nd	rate (AE:138. 8, CE: 125.4)				Digit Span	
Kvalø et al. (2017)	449 (227/222)	230 vs. 219	9.6±0.7 vs. 9.3±0.6	N/A	ME: Running to answer questions, Capture the Flag, Tag, Jump Rope, Strength Training.	Regular PE lessons	N/A	N/A	65 min	2 × per week	40 weeks	Postintervent ion, Digit Span Test	
Latino et al. (2021)	60 (30/30)	30 vs. 30	14.4±0.5	21.4±2.0	ME: Includes Slalom circuits, Jump rope exercises, Rhythm exercises, Motor responses exercises, etc.	AE: Includes group exercises , jogging, calistheni cs workout, Pilates, etc.	Indoor Gymnasi um	Medium-high intensity	25 min	2 × per week	12 weeks	Postintervent ion, Corsi's Block-Tappi ng Test	
Leahy et al. (2020)	62 (33/29)	18/15 vs. 14/15	16.2±0.4 vs. 16.2±0.4	N/A	HIIT: Combination of aerobic exercise and resistance exercises (e.g., star jumps, squat jumps)	Usually activities	Indoor Classroo m	work-to-rest ratio (30s:30s)	12-20 min	3 × per week	14 weeks	Postintervent ion, N-back task	Postintervention , N-back task
Leong et	81 (41/40)	0/41 vs. 0/40	15.7±0.6 vs.	N/A	DC: Aerobic dance.	Not	N/A	70% of	60 min	2 × per	6 weeks	Postintervent	

al. (2015)			15.3±0.8			receive any intervention		maximum heart rate		week		ion, Digit Span Test	
Lind et al. (2017)	931 (838/93)	411/427 vs. 41/52	11.9±0.4 vs. 11.8±0.2	18.4±3.2 vs. 19.3±3.7	SG: Football games and playing small-sided football.	Usually activities	Outdoor Playground	N/A	45 min	2 × per week	11 weeks		Postintervention, N-back task
Lubans et al. (2020)	670 (337/333)	168/169 vs. 203/130	16.0±0.4 vs. 16.0±0.5	N/A	HIIT: Combination of aerobic exercise and self-weighted resistance exercises (e.g., round-trip running, open-close jumps, push-ups)	Usually activities	Indoor Classroom	85% of maximum heart rate	8-20 min	2 × per week	6 months	Postintervention, N-back task	Postintervention, N-back task
Ludyga et al. (2018)	33 (17/16)	13/4 vs. 8/8	12.5±0.7 vs. 12.4±0.7	18.2±2.2 vs. 19.7±3.6	SG: Combination of aerobic and coordinated games, e.g. relay games, ball games.	Talks with classmates	Outdoor Playground	Average heart rate (134.6)	20 min	5 × per week	8 weeks	Postintervention, Sternberg task	Postintervention, Sternberg task
Mavilidi et al. (2020)	58 (29/29)	14/15 vs. 20/9	8.7±0.5 vs. 9.2±0.6	N/A	HIIT: Combat (straight and cross-over punches, squats), fitness (skipping, jumping jacks, jogging on the spot), and cardio (lunges, skater jumps, push ups).	Mathematical activities	Indoor Classroom	4 METs	5 min	3 × per week	4 weeks	Postintervention, N-back task	
Meijer et al. (2021)	621 (206/235/4	102/104 vs. 109/126 vs.	9.3±0.7 vs. 9.0±0.6 vs.	16.8±2.1 vs. 16.8±2.3 vs.	(a) AE: Circuit training, relay games, playing tag, and	Regular PE	Outdoor Playgrou	(a) AE: 12.25±3.	20 min	(a) AE: 4 × per week	14 weeks	Postintervention, Digit	

	15)	209/206	9.2±0.7	16.5±2.3	individual activities like running.<br>(b) SG: Team games or exercises include dodgeball, basketball, or soccer.	lessons	nd	03 MVPA per lesson (min)<br>(b) SG: 9.45±2.52 MVPA per lesson (min)		(b) SG: 4 × per week<br>(c) CON: 2 × per week		Span	
Meijer et al. (2022)	93 (30/32/31)	15/15 vs. 16/16 vs. 15/16	9.2±0.7 vs. 9.1±0.6 vs. 9.2±0.6	16.7±2.1 vs. 17.0±2.6 vs. 16.6±1.9	(a) AE: Circuit training, relay games, playing tag, and individual activities like running.<br>(b) SG: Team games or exercises include dodgeball, basketball, or soccer.	Regular PE lessons	Outdoor Playground	Moderate-to-vigorous intensity	20 min	(a) AE: 4 × per week<br>(b) SG: 4 × per week<br>(c) CON: 2 × per week	14 weeks	Postintervention, Digit Span	
Mora-Gonzalez et al. (2023)	67 (35/32)	24/11 vs. 17/15	10.0±1.1 vs. 10.1±1.1	27.1±4.1 vs. 26.1±2.9	ME: (a) 4-5 moderate to high intensity cardio games (60 min);<br>(b) Resistance training for muscle and bone strengthening (20 min).	Usually activities	Outdoor Playground	Moderate-to-vigorous intensity	80 min	3 × per week	20 weeks	Postintervention, Delayed non-matched-to-sample task	Postintervention, Delayed non-matched-to-sample task
Park et al.	66	0/22 vs. 0/22 vs.	14.4±1.0 vs.	23.3±5.7 vs.	(a) DC: Stepping to the	Not	N/A	RPE	25 min	3 × per	12 weeks	Postintervent	

(2022)	(22/22/22)	0/22	14.7±0.8 vs. 14.8±0.8	19.8±5.9 vs. 20.6±8.2	rhythm of music; (b) AE: Stepping without musical rhythm, following a set pattern.	receive any intervention		(12–14)		week		ion, Word list memory test	
Robinson et al. (2022)	73 (29/23)	12/17 vs. 15/8	15.9±0.4 vs. 15.8±0.4	N/A	HIIT: Resistance training in Tabata mode, 20 seconds of work/10 seconds of rest × 8 sets, total workout time 4 minutes	Sedentary control	Indoor Classroom	Borg scale (CR-10) (2.25)	4 min	3 × per week	4 weeks	Postintervention, Picture sequence memory test	
Schmidt et al. (2015)	181 (69/57/55)	26/43 vs. 28/29 vs. 28/27	11.3±0.6 vs. 11.3±0.6 vs. 11.4 ± 0.6	18.2±2.8 vs. 17.4±2.5 vs. 17.6±2.6	(a) SG: Team games including tag, floorball and basketball. (b) AE: Different group-oriented and playful forms of aerobic exercises.	Regular PE lessons	Outdoor Playground	Average heart rate (SG:147.9, AE: 150.3)	45 min	2 × per week	6 weeks	Postintervention, N-back task	
St Laurent et al. (2019)	53 (27/26)	18/9 vs. 11/15	8.8±0.1 vs. 9.4±0.1	N/A	SG: 25-40% muscular fitness and 60-75% cardiorespiratory bingo games.	Standard practice	N/A	Average heart rate (122.5)	13 min	5 × per week	3 months	Postintervention, List Sorting Working Memory Test	
Tocci et al. (2022)	95 (46/49)	23/23 vs. 24/25	7.7±1.2 vs. 7.8±1.4	17.8±2.9 vs. 18.0±3.0	ME: Spell out letters and numbers physically or play board games and jigsaw puzzles; perform dance routines to music.	Not receive any intervention	N/A	N/A	60 min	1 × per week	24 weeks	Postintervention, Random Number Generation	

Torbeyns et al. (2017)	44 (21/23)	8/13 vs. 13/10	14.3±0.6	19.7±3.5 vs. 20.1±3.7	AE: Cycling on a height-adjustable bike table (LifeSpan C3-DT5 Bike Table).	Not receive any intervention	Indoor Classroom	Borg scale (CR-10) (6.2)	40 min	5 × per week	15 weeks	Postintervention, Rey Auditory Verbal Learning Test	
Tottori et al. (2019)	56 (27/29)	17/10 vs. 14/15	10.0±1.0 vs. 10.4±1.1	16.9±2.7 vs. 16.3±1.8	HIIT: Aerobic and core exercise using one's own weight.	Not receive any intervention	Indoor Gymnasium	Average heart rate (170.0), work-to-rest ratio (30s:30s)	8-10 min	3 × per week	4 weeks	Postintervention, Backward digit span	
van den Berg et al. (2019)	512 (263/249)	142/121 vs. 132/117	10.8±0.6 vs. 10.9±0.7	17.2±2.3 vs. 17.3±2.3	DC: Consists of three "Just Dance" videos in which children are asked to imitate dancing.	Educational lessons	Indoor Classroom	60% of maximum heart rate	10 min	5 × per week	9 weeks	Postintervention, Fluency Task	
Veldman et al. (2020)	60 (30/30)	16/14 vs. 14/16	7.6±1.8 vs. 7.8±1.9	N/A	SG: Consists of team game sports that rotate between football, netball, basketball and touch/oztag, or themed sessions based on popular TV shows such as The Amazing Race and Survivor.	Usually activities	After-school Care Centers	N/A	75 min	3 × per week	24 weeks	Postintervention, Mr. Ant task.	
Wassenaar	16017(7860	3394/4466 vs.	12.5±0.3 vs.	N/A	HIIT: Aerobics consist of	Regular	N/A	71-85%	10 min	2 × per	10	Postintervent	Postintervention

et al. (2021)	/8157)	3662/4495	12.5±0.3		squats and lunges, and sprinting on the spot.	PE lessons		of maximum heart rate, work-to-rest ratio (10s:50s)		week	months	ion, Visual two-back task	, Visual two-back task
Zhang et al.(2023)	100 (49/50)	49 vs. 50	6.2±0.3 vs. 6.1±0.3	16.2±1.8 vs. 17.2±2.7	AE: Running, stretching, jumping rope, sport games.	Usually activities	Outdoor Playground	60-70% of maximum heart rate	30 min	4 × per week	11 weeks	Postintervention, Working memory span task	
Zinelabidine et al. (2022)	41 (19/22)	10/9 vs. 12/10	10.3±0.6 vs. 10.0±0.4	17.4±3.1 vs. 19.2±5.5	DC: Continuous aerobic dance exercises.	Regular PE lessons	Indoor Aerobic Room	Average heart rate (130)	30 min	2 × per week	8 weeks	Postintervention, Digit Recall Test	

Note: BMI, Body Mass Index; AE, Aerobic Exercise; CON, Control Group; DC, Dance; HIIT, High-Intensity Interval Training; ME, Mixed Exercise; SG, Sports Games; N/A, Not Applicable.

Supplementary 5: Risk of Bias

Author	Bias arising from the randomization process	Bias due to deviations from intended intervention	Bias due to missing outcome data	Bias in measurement of the outcome	Bias in selection of the reported result	Overall
Alesi et al. (2016)	Low	Some concerns	Low	Low	Low	Some concerns
Beck et al. (2016)	Some concerns	Low	Low	Low	Low	Some concerns
Contreras-Osorio et al. (2022)	Low	Low	Low	Low	Low	Low
de Bruijn et al. (2021)	Low	Low	Some concerns	Low	Low	Some concerns
Egger et al. (2019)	Low	Low	Low	Low	Low	Low
Gentile et al. (2020)	Some concerns	Some concerns	Some concerns	Low	Low	Some concerns
Jeon et al. (2017)	Low	Low	Low	Low	Low	Low
Kamijo et al. (2011)	Some concerns	Low	Low	Low	Low	Some concerns
Knatauskaitė et al. (2021)	Low	Low	Low	Low	Low	Low
Koutsandréou et al. (2016)	Low	Low	Low	Low	Low	Low
Kvalø et al. (2017)	Low	Low	Some concerns	Low	Low	Some concerns
Latino et al. (2021)	Low	Low	Low	Low	Low	Low
Leahy et al. (2020)	Low	Low	Low	Low	Low	Low
Leong et al. (2015)	Low	Low	Low	Low	Low	Low
Lind et al. (2017)	Low	Some concerns	Low	Some concerns	Low	Some concerns
Lubans et al. (2020)	Low	Low	High	Low	Low	High

Ludyga et al. (2018)	Low	Low	Low	Low	Some concerns	Some concerns
Mavilidi et al. (2020)	Low	Low	Low	Low	Low	Low
Meijer et al. (2021)	Low	Low	Some concerns	Low	Low	Some concerns
Meijer et al. (2022)	Low	Low	Some concerns	Low	High	High
Mora-Gonzalez et al. (2023)	Low	Low	Low	Low	Low	Low
Park et al. (2022)	Low	Low	Low	Low	Low	Low
Robinson et al. (2022)	Low	Low	Some concerns	Low	Low	Some concerns
Schmidt et al. (2015)	Low	Low	Low	Low	Low	Low
St Laurent et al. (2019)	Some concerns	Some concerns	Some concerns	Low	Low	Some concerns
Tocci et al. (2022)	Low	Low	Low	Low	Low	Low
Torbeyns et al. (2017)	Some concerns	Low	Low	Low	Low	Some concerns
Tottori et al. (2019)	Low	Low	Low	Low	Low	Low
van den Berg et al. (2019)	Low	Low	Low	Low	Low	Low
Veldman et al. (2020)	Low	Low	High	Low	Low	High
Wassenaar et al. (2021)	Low	Low	Low	Low	Low	Low
Zhang et al.(2023)	Some concerns	Low	Low	Low	Low	Some concerns
Zinelabidine et al. (2022)	Low	Low	Low	Low	Low	Low

Supplementary 6: Publication bias

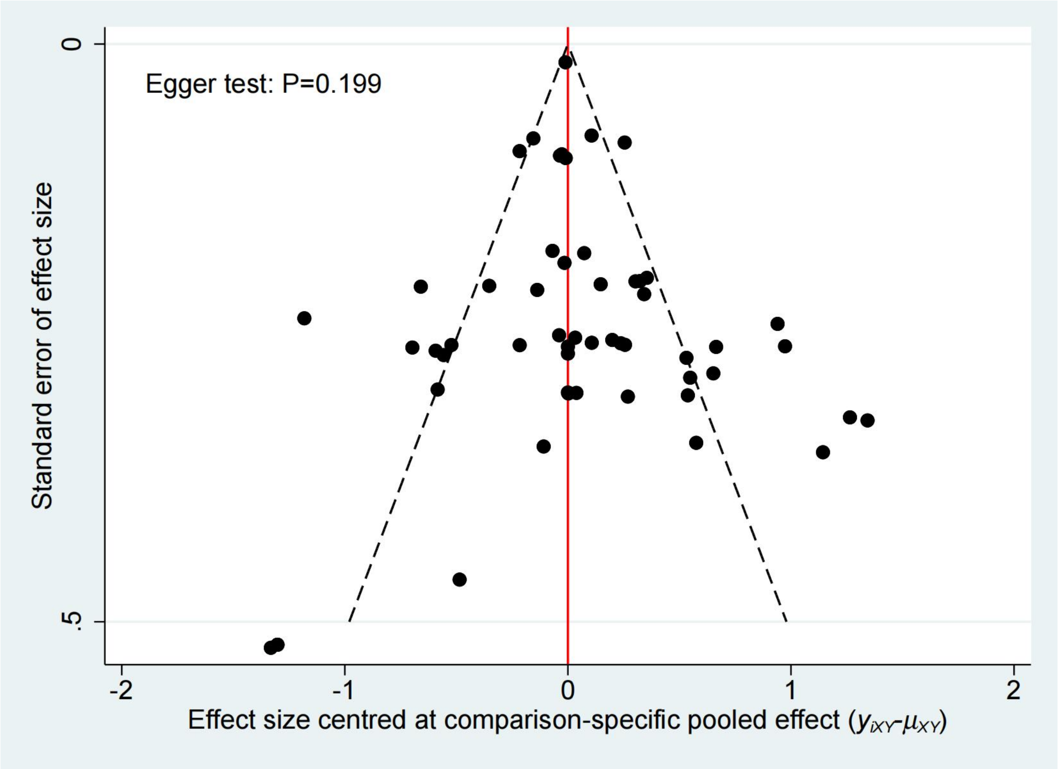

Figure 6.1 The funnel plot of working memory accuracy. The result of Egger test showed the p=0.199.

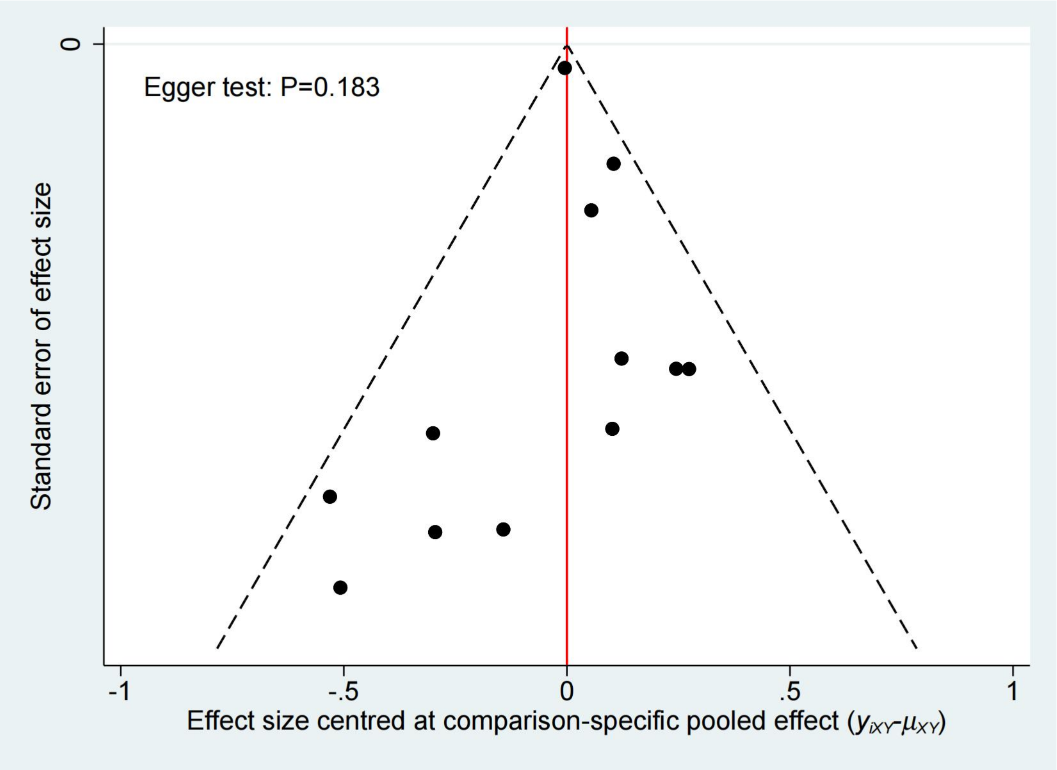

Figure 6.2 The funnel plot of working memory reaction time. The result of Egger test showed the p=0.183.
